# Concurrent Growth Rate and Transcript Analyses Reveal Essential Gene Stringency in *Escherichia coli*

**DOI:** 10.1371/journal.pone.0006061

**Published:** 2009-06-26

**Authors:** Shan Goh, Jaroslaw M. Boberek, Nobutaka Nakashima, Jem Stach, Liam Good

**Affiliations:** 1 Department of Cell and Molecular Biology, Karolinska Institute, Stockholm, Sweden; 2 Department of Pathology and Infectious Diseases, Royal Veterinary College, University of London, London, United Kingdom; 3 Research Institute of Genome-based biofactory, Toyohira-Ku, Sapporo, Japan; 4 School of Biology, University of Newcastle, Newcastle upon Tyne, United Kingdom; BMSI-A*STAR, Singapore

## Abstract

**Background:**

Genes essential for bacterial growth are of particular scientific interest. Many putative essential genes have been identified or predicted in several species, however, little is known about gene expression requirement stringency, which may be an important aspect of bacterial physiology and likely a determining factor in drug target development.

**Methodology/Principal Findings:**

Working from the premise that essential genes differ in absolute requirement for growth, we describe silencing of putative essential genes in *E. coli* to obtain a titration of declining growth rates and transcript levels by using antisense peptide nucleic acids (PNA) and expressed antisense RNA. The relationship between mRNA decline and growth rate decline reflects the degree of essentiality, or stringency, of an essential gene, which is here defined by the minimum transcript level for a 50% reduction in growth rate (MTL_50_). When applied to four growth essential genes, both RNA silencing methods resulted in MTL_50_ values that reveal *acpP* as the most stringently required of the four genes examined, with *ftsZ* the next most stringently required. The established antibacterial targets *murA* and *fabI* were less stringently required.

**Conclusions:**

RNA silencing can reveal stringent requirements for gene expression with respect to growth. This method may be used to validate existing essential genes and to quantify drug target requirement.

## Introduction

Progress in antimicrobial discovery has been slow during recent decades [1] despite large-scale efforts to identify genes essential for growth in conserved pathways of *Escherichia coli* [2] and *Staphylococcus aureus* [3]. The drug discovery process involves essential gene identification through various methods such as chromosomal deletions [2] and experimentally reduced genomes [4], followed by studies of gene product interactions through tandem affinity purification [5] and/or mathematical models [6]. Potential drug targets are then subjected to high-throughput screening for inhibitors, which often fail at the cellular level in “hit-to-lead” stages of development due to several factors [7], such as gene function redundancy [8]. Also, for certain essential genes we suspect that there may be a low degree of gene expression requirement for bacterial viability. Despite evidence for differential requirement in growth essential genes [9], and initial efforts at scoring essentiality [2], [10], there has not been a quantitative method to determine the expression requirement for essential genes so that only stringently required targets are considered further for drug development. This study focuses on measuring differences between suspected essential genes in terms of their degree of requirement for cell viability, referred to here as stringency, in *E. coli*.

We hypothesize that growth essential genes differ in requirement stringencies, and that these differences can be revealed by measuring the relationships between specific mRNA decreases and bacterial growth rate decline. The aim of this study is to measure the growth stringency requirement *E. coli* genes using antisense gene silencing, so as to evaluate degree of essentiality for cell viability. Two antisense gene control strategies for bacteria are used - synthetic antisense peptide nucleic acids (PNAs) and plasmid expression of antisense RNA sequences. Both methods are capable of modulating essential genes *in situ* in *E. coli* [11], [12] and *S. aureus* [13], [14], and certain antisense agents can inhibit Mycobacteria [15], [16]. Therefore, RNA silencing using specific antisense sequences provides an approach to measure gene requirement stringency.

Four genes, *acpP*, *fabI*, *ftsZ* and *murA* were selected for this study for the following reasons: (a) each gene has been shown experimentally to be essential for growth in *E. coli* [2], [4], [17], [18], [19], [20], (b) they have been studied as antimicrobial targets [21], [22], [23], (c) the gene products have different cellular functions and (d) each gene can be silenced potentially without downstream effects [24], [25]. Interactions between FabI and its specific inhibitor, triclosan, are well understood [26]. The *fabI* gene encodes enoyl ACP reductase, which catalyzes fatty acid elongation [17]. Another well-studied drug target is UDP-N-acetylglucosamine enolpyruvyl transferase (encoded for by *murA*), which catalyzes the synthesis of peptidoglycan from N-acetylglucosamine acid and phosphoenoylpyruvate [10], [18], [27]. MurA is specifically inhibited by phosphomycin [28], and the mechanism of inhibition is well understood [29]. The protein function of FtsZ has also been studied in detail, and FtsZ is the focus of much assay development to discover effective inhibitors [30]. The *ftsZ* gene encodes a tubulin-like protein that polymerizes to form a Z-ring as a scaffold for cell division [31]. ACP or acyl carrier protein is encoded by *acpP*. ACP is an interesting drug target because, although it has been shown to be central to bacterial fatty acid biosynthesis [32], [33], specific protein inhibitors have not yet been discovered. Nevertheless, *acpP* was shown to be an effective antisense target for treatment of bacterial infections *in vivo* [34], [35] and the gene product can be inhibited [36].

In principle, the extent to which partial mRNA inhibition limits growth should reflect how stringently required the targeted mRNA is for bacterial viability. The specificities of antisense PNA and expressed antisense RNA silencing of the four selected target genes were assessed by conducting rescue experiments, where over-expression of the four target genes in different strains complemented the effects of the cognate silencers. To titrate down essential gene expression and obtain measured responses in *E. coli*, growth, we used gene specific antisense PNA and IPTG-induced expression of antisense sequences. This concept enables quantitative analyses of mRNA silencing and growth reduction, which are not possible using knockout approaches. We show that the essential genes differed in mRNA inhibition to growth inhibition profiles, demonstrating that the relationship between transcript levels and growth rate reflects the requirement stringencies of essential genes.

## Results

### Specificity of antisense RNA PNA and expressed antisense RNA

**Figure 1:**
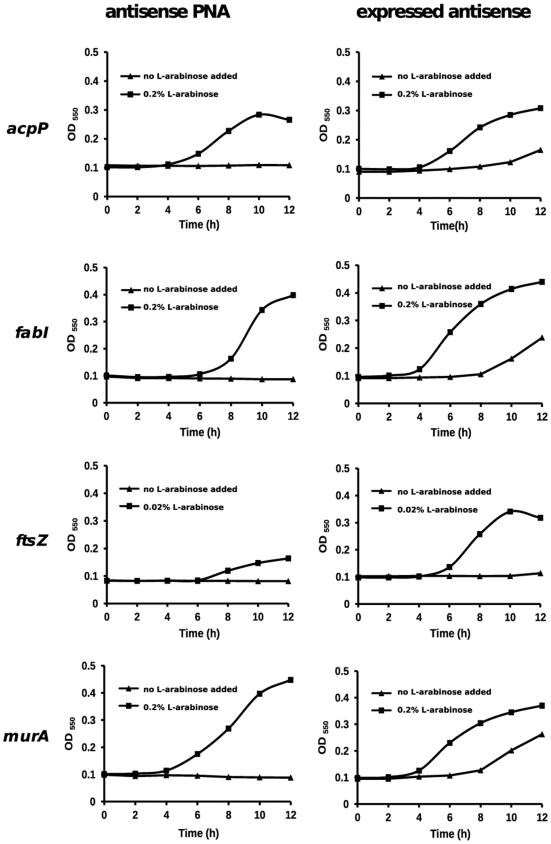
Transcomplementation of antisense PNA and expressed antisense RNA effects on growth by target gene over-expression. Essential gene over-expression was induced by L-arabinose at indicated concentrations. PNAs were added to a final concentration of 4 µM. Concentrations of IPTG used for induction of *acpP*-, *fabI*-, *ftsZ*- and *murA*-antisense expression were 200 µM, 1 mM, 100 µM and 75 mM, respectively.

To silence the four selected target genes using the two different silencing methods, we first needed to design, test and validate several new silencers. Fortunately, we were able to use previously developed silencers for *acpP* and *fabI*, and we used similar design guidelines for the additional silencers needed for this study. We found that all eight silencers are able to inhibit *E. coli* growth. Also, both PNA and expressed antisense RNA silencers displayed gene and sequence selective effects. Nevertheless, to ensure that all silence mainly the target gene, we carried out a set of rescue experiments where the RNA silencers were used under conditions that inhibited growth and we tested whether growth could be rescued by over-expression of the target gene open reading frame (ORF) from a plasmid. This transcomplementation strategy provides a more strict control of specificity relative to controls that involve sense, scrambled or irrelevant sequences, because it takes into account all transcript sequences present in the bacterial cell and the effector sequences remain unchanged. We designed antisense PNA specific for *acpP* (Ec108), *fabI* (Ec107), *ftsZ* (Ec326), and *murA* (Ec330) using optimal parameters for gene silencing [37], [38] (Table 1). To test PNA specificity, we constructed four DH5α strains containing either pBAD-acpP, pBAD-fabI, pBAD-ftsZ or pBAD-murA. Each strain was grown in the presence of antisense PNA specific to the target gene cloned into pBAD, and either with or without the addition of L-arabinose for target gene over-expression. Without L-arabinose, strains did not grow but upon induction by L-arabinose, were resistant to the growth inhibitory effects of the PNA (Figure 1). To test specificity of antisense expressed from plasmids, we constructed another four DH5α strains containing either pBAD-acpP+pHNA, pBAD-fabI+pHN682, pBAD-ftsZ+pHNZ or pBAD-murA+pHNM. Each strain was grown in the presence of IPTG for induction of expressed antisense, and either with or without the addition of L-arabinose to induce target gene over-expression. Without L-arabinose, growth of strains was inhibited, and L-arabinose addition complemented gene silencing and allowed strains to grow in the presence of expressed antisense (Figure 1). The results from the rescue experiments demonstrate that the eight silencers display gene selective growth inhibition.

**Table 1:** Properties of PNA used in this study.

PNA	Sequence	Target	Target location* and length	Reference/source
Ec107	(KFF)_3_K-eg1-cccatagctt	*fabI*	−5 to +5 (10 nt.)	[11]
Ec108	(KFF)_3_K-eg1-ctcatactct	*acpP*	−5 to +5 (10 nt.)	[57]
Ec326	(KFF)_3_K-eg1-tcaaacatag	*ftsZ*	−2 to +8 (10 nt.)	This study
Ec330	(KFF)_3_K-eg-1-tttagtttgt	*murA*	−9 to +1 (10 nt.)	This study

* Antisense target locations are indicated relative to the start codon.

### Titration of essential gene expression and its effect of growth rate

**Figure 2:**
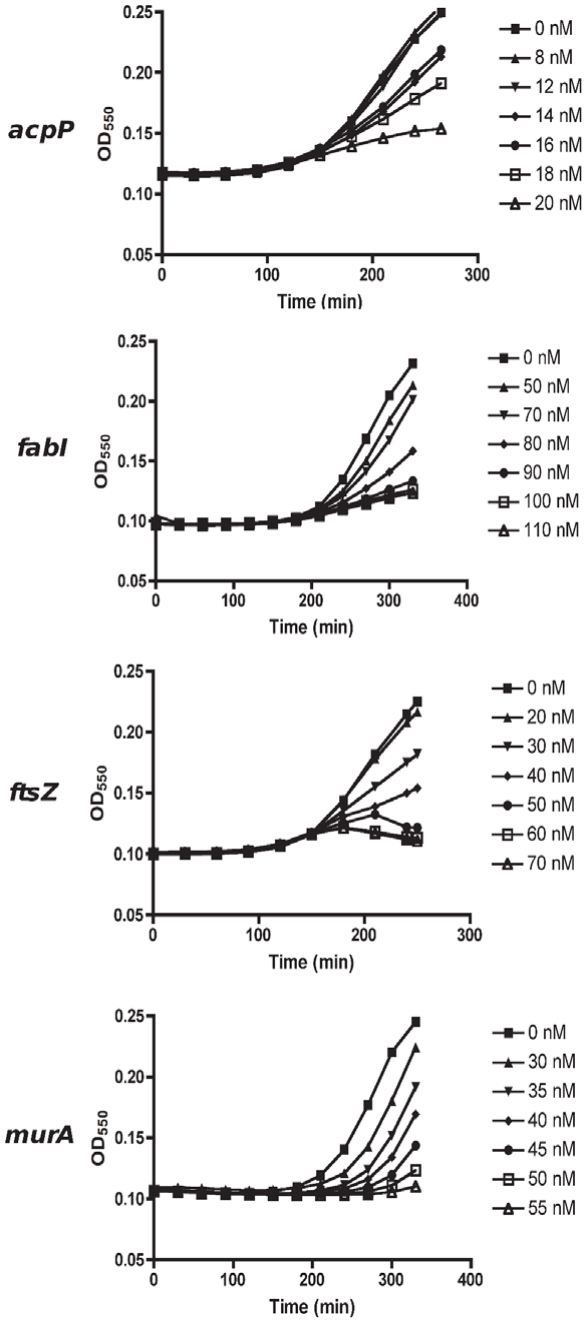
Dose dependent growth inhibition of antisense PNA-treated AS19 cells. Overnight AS19 cultures were sub-cultured in fresh media containing different PNA doses for each gene, and its growth monitored by turbidity. Six doses were chosen for each PNA, so as to obtain a titration in growth inhibition. Growth profiles terminated when all cultures were harvested at ΔOD_550_∼0.1 of the untreated culture, for transcript analyses.

To test the hypothesis that essential genes are differentially required to maintain a similar level of growth, we titrated down the growth rate of *E. coli* using either antisense PNA or expressed antisense and determined mRNA levels of the targeted essential gene. To enable parallel analyses of multiple genes in this study, we used RT-PCR to quantify mRNA levels and assume it as a proxy to protein measure in bacteria [39]. For PNA-mediated antisense effects, we used the hyper-permeable *E. coli* strain AS19 to obtain efficient uptake [40]. Six doses of each antisense PNA were selected in order to achieve a titration in *E. coli* growth rate reduction. Hence, the dose range of each antisense PNA was unique, resulting in growth inhibition in a dose dependent manner (Figure 2). For expressed antisense, plasmids were transformed into TOP10 *E. coli* cells (Table 2) and antisense RNA expression was induced using IPTG at concentrations that provided a titration of decreasing growth rates (not shown).

**Table 2:** Plasmids used in this study.

Plasmid	Relevant characteristic	Purpose	Antisense target location* and length	Reference/source
pBAD/HisA	Arabinose inducible promoter (P_BAD_), Amp^R^	Inducible expression of essential genes	n.a.	Invitrogen
pBAD-fabI	*fabI* ORF	Inducible over-expression of *fabI*	n.a	This study
pBAD-acpP	*acpP* ORF	Inducible over-expression of *acpP*	n.a.	This study
pBAD-ftsZ	*ftsZ* ORF	Inducible over-expression of *ftsZ*	n.a	This study
pBAD-murA	*murA* ORF	Inducible over-expression of *murA*	n.a.	This study
pHN678	IPTG inducible promoter (P_trc_), paired-termini flanking MCS, Cam^R^	Stabilized antisense expression vector	n.a.	[12]
pHN682	*fabI* antisense insert	Inducible expression of *fabI* antisense	−74 to +86 of *fabI* (160 nt.)	[12]
pHNA	*acpP* antisense insert	Inducible expression of *acpP* antisense	−42 to +85 of *acpP* (127 nt.)	This study
pHNZ	*ftsZ* antisense insert	Inducible expression of *ftsZ* antisense	−53 to +76 of *ftsZ* (129 nt.)	This study
pHNM	*murA* antisense insert	Inducible expression of *murA* antisense	−54 to +76 of *murA* (130 nt.)	This study

* Antisense target locations are indicated relative to the start codon.

**Figure 3:**
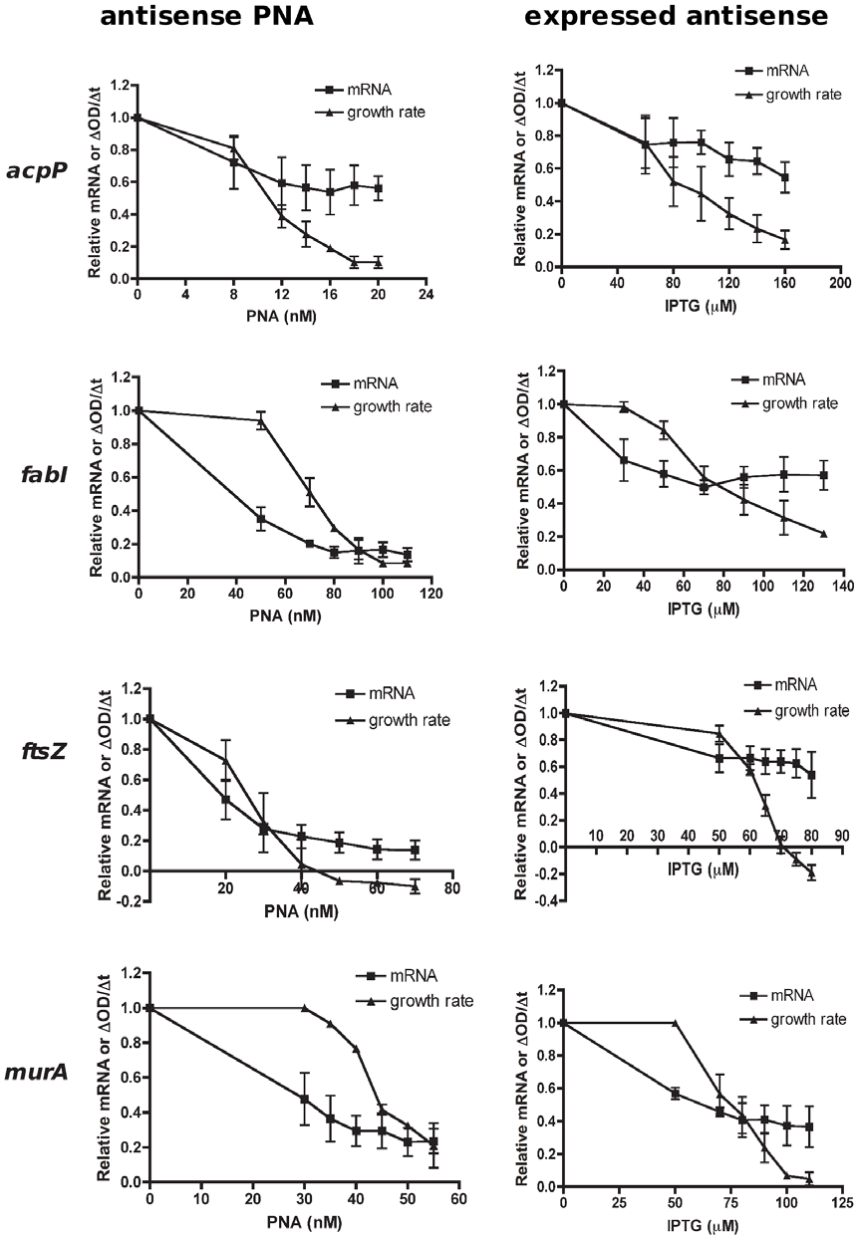
Gene silencing in *E. coli* to obtain a titration of declining growth rates and transcript levels by antisense PNA and expressed antisense. *E. coli* AS19 was treated with PNA doses, while TOP10 clones containing antisense-expressing plasmids were induced with various IPTG concentrations as shown in Figure 2, so as to obtain growth inhibition in a dose-dependent manner. Total RNA from *E. coli* cultures was harvested at the time when the control culture increased in OD_550_ by 0.1. RNA was used for cDNA synthesis and quantitative real-time PCR to determine mRNA levels by normalization to a reference gene *rpoA*, and then calculated relative to the unsilenced (untreated) control. Growth rates were determined by OD_550_ readings over time. The relationship between mRNA and growth rate decline indicated growth requirement stringency of each gene.

**Figure 4:**
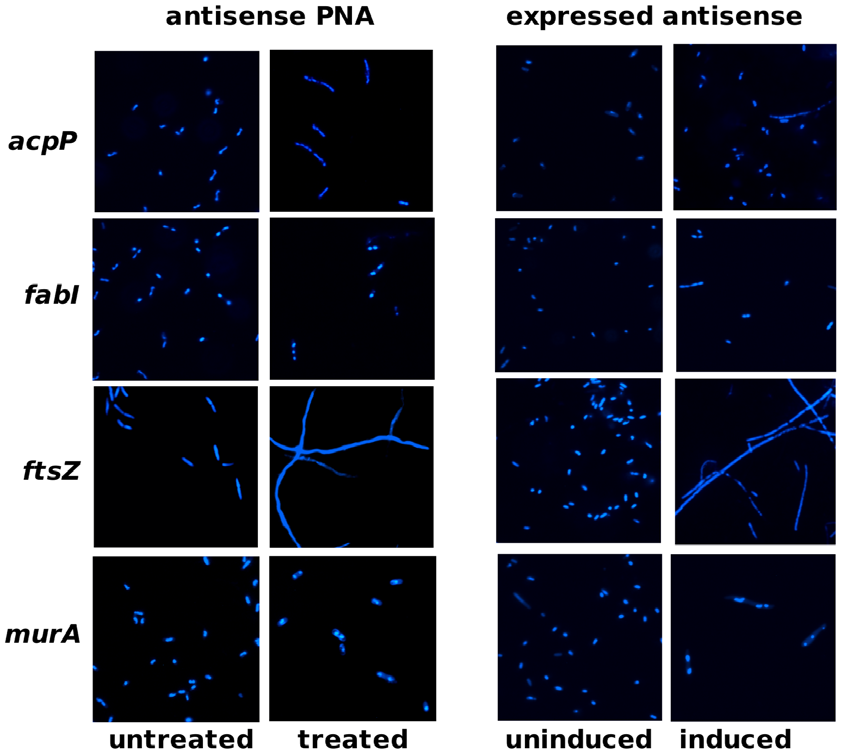
Effect of gene silencers on cell morphology. Cells were stained with DAPI before fluorescent microscopy. Left panel: AS19 untreated or treated with antisense PNA Ec108 (20 nM) targeting *acpP*, Ec107 (80 nM) targeting *fabI*, Ec326 (70 nM) targeting *ftsZ* or Ec330 (55 nM) targeting *murA*. Gross elongation of cells was observed only in cultures treated with *ftsZ*-specific PNA, Ec326. Right panel: TOP10 clones uninduced or induced with IPTG for antisense RNA expression. Concentrations of IPTG used for induction of *acpP*-, *fabI*-, *ftsZ*- and *murA*-antisense expression were 160 µM, 130 µM, 80 µM and 80 µM, respectively. Cells expressing *ftsZ*-antisense were grossly elongated compared to other cells.

Both antisense PNA and expressed antisense specifically silenced mRNA of essential genes *acpP*, *fabI*, *ftsZ* or *murA*, as determined by analyses of transcript levels over an appropriate range of inhibitory but not lethal doses (Figure 3). Decreases in essential gene transcripts corresponded to decreases in bacterial growth rates (PNA data: R^2^ for *acpP*, *fabI*, *ftsZ*, *murA* = 0.87, 0.63, 0.89, 0.45), although the response was non-linear. It should be noted that the growth profiles observed for *ftsZ*-inhibited cultures, which displayed a rise and then a fall in culture turbidity, were consistent with a cell-division effect observed previously, where cells elongate without dividing [41], [42]. Phenotypic effects of *ftsZ*-inhibited cultures were confirmed by microscopy, revealing extremely elongated cells compared to untreated cells and cells treated with other PNAs. Cell phenotypes for antisense-expressing cells were similar to that of antisense PNA-treated cells (Figure 4).

### Essential gene stringencies and minimum level transcript 50 as a measure

**Figure 5:**
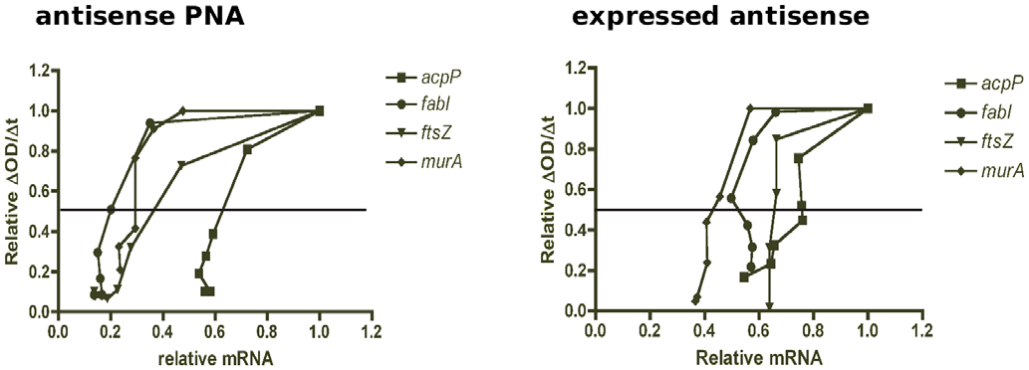
Determining the MTL_50_ value of each gene. For each gene, mean relative mRNA and growth rate values from Figure 3 were transformed so that mean relative mRNA values were plotted against corresponding mean values of relative growth rates to obtain a curve. The curve of each gene allows an estimation of a transcript value (on the x-axis) at a growth reduction of 50% (y = 0.5), that is, the MTL_50_ value of the gene of interest. The MTL_50_value reveals stringency of an essential gene for growth.

The relationship between the decrease in mRNA and growth rate determined by both silencing methods indicates that the requirement for the four essential genes is unequal, with some genes more stringently required than others. Indeed, expressing relative growth rate as a function of relative mRNA level (Figure 5) showed that the minimum level of mRNA needed for 50% cell viability is in the order *acpP* >*ftsZ*>*murA*>*fabI* by antisense PNA. That is, a large number of *acpP* transcripts (approximately 0.65 relative value or 65% of a normal cell) is required to maintain 50% cell viability, compared to *ftsZ* (38%), *murA* (30%) and *fabI* (20%) transcripts. In other words, a small decrease in *acpP* mRNA led to a large decrease in bacterial growth rates, while decreases in *ftsZ* mRNA had proportional effects on growth rates and large decreases in *fabI* and *murA* mRNA were required to obtain a small decrease in growth rates. Gene stringency was in the order *acpP*>*ftsZ*>*fabI*>*murA* by expressed antisense (Figure 5), which differs from the PNA data in that *murA* is the least stringently required essential gene for growth rather than *fabI*.

**Figure 6:**
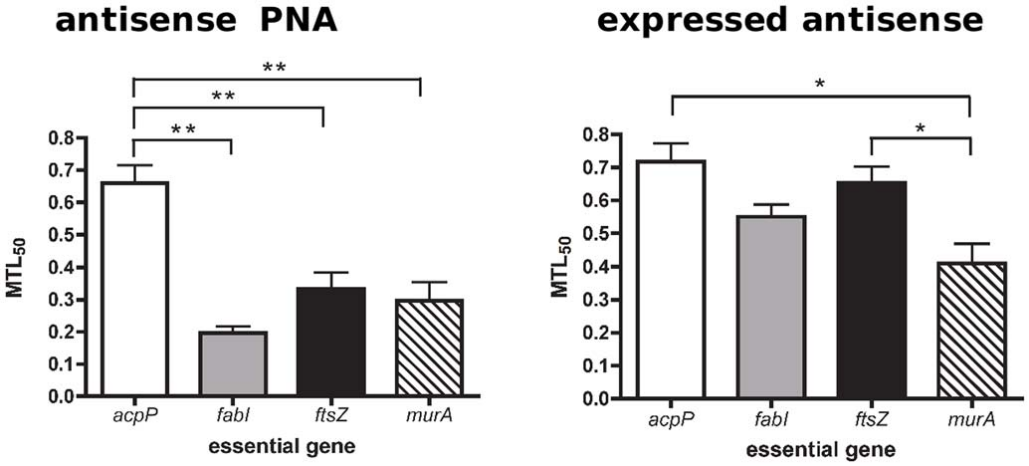
Analysis of essential gene MTL_50_ values. Three curves from triplicate experiments of each gene were plotted so as to obtain three MTL_50_ values for statistical analyses. Significant difference (** = p<0.01, * = p<0.05) in MTL_50_ between genes was determined by a one-way ANOVA and Tukey HSD Test. Antisense PNA silencing (left panel) showed MTL_50_ of *acpP* to be significantly greater than the other three genes. Expressed antisense RNA silencing (right panel) showed MTL_50_ of *acpP* and *ftsZ* was significantly greater than that of *murA* only.

Based on the above data, we propose minimum transcript level 50 (MTL_50_) as a measure of stringent requirement, somewhat similar to the IC_50_ measure of enzyme inhibition. MTL_50_ values directly reflect the relationship between the level of a particular mRNA and growth rate decline, thus indicating how stringently required an essential gene is for growth. By plotting the values of relative growth rate against relative mRNA level for each gene on the same graph, we are able to estimate the value of relative mRNA (MTL_50_) at 50% growth rate (Figure 5). We observed that a shift to the right indicates greater stringency as the minimum level of transcript needed to sustain 50% cell viability increases. Hence, a high MTL_50_ value indicates high gene stringency. This is because the value indicates the minimum required level of transcript for viability, not the minimum inhibition level of transcript, and hence differs from IC_50_ in this respect. Comparisons of the MTL_50_ values, determined by the two different gene-silencing methods, are shown in Figure 6. In general, MTL_50_ values were higher in the expressed antisense cultures compared to antisense PNA treated cultures. Both methods found that the most stringently required essential gene was *acpP*, followed by *ftsZ*. Antisense PNA silencing showed *fabI* to be the least stringent, whereas expressed antisense silencing found *murA* was significantly less stringently required, relative to the other four essential genes examined (Figures 5 and 6).

## Discussion

Cell growth and mRNA profiles following expressed antisense RNA silencing differed somewhat from those following antisense PNA silencing, possibly reflecting different mechanisms of silencing. Nevertheless, the data reject the null hypothesis of equal stringent requirement for growth essential genes and support our premise that essential genes differ in their level of requirement for growth. Indeed, both RNA silencing methods indicate significant differences in gene stringency and reveal *acpP* as the most stringently required essential gene, followed by *ftsZ*, out of the four that were studied. As the MTL_50_ value of each gene is calculated relative to appropriate unsilenced controls, differences in basal expression of the different essential genes will not affect the stringency ranking.

The stringency ranking of *murA* and *fabI* differed between PNA-treated cells and antisense-expressing cells, probably because of differences between PNA and RNA chemistry, size differences between the two types of silencers and subtle differences in the mechanisms of gene silencing. For example, antisense PNAs act directly through steric hindrance of translation initiation and may indirectly trigger decay of repressed transcripts, whereas antisense RNAs may act both by steric hindrance and by direct activation of dsRNA-mediated cleavage at the target site. Ideally, protein quantification should be carried out in parallel with RT-PCR, however, biochemical assays to confirm antisense inhibition of *acpP*, *fabI*, *ftsZ* and *murA* were not carried out due to the technical difficulties involved in harvesting sufficient culture biomass and quantitative protein assays are lacking for many genes of interest. Current methods are limited to the use of antibodies [33], [43] or intracellular peptide expression [44]. Nevertheless, correlation between mRNA and protein levels in bacteria has been demonstrated for some genes [25], [39], [45], [46], [47], suggesting that the MTL values reported here provide a useful measure of gene requirement.

Four genes were compared in this study and only *acpP* does not encode for an enzyme. However, the finding of *acpP* being more critically required than *ftsZ*, *fabI* and *murA* is not entirely surprising. ACP is known to interact with a large number of proteins in the fatty acid biosynthesis pathway [5] and reduction in levels of active ACP results in cell toxicity [33], [36]. In addition, ACP interacts with proteins not involved in lipid synthesis [48]. Therefore, ACP is needed in large quantities and a slight decrease in ACP levels may be amplified through its interacting partners, leading to widespread physiological effects. Indeed, *acpP* transcripts and protein products are abundant in *E. coli* [49], [50]. FtsZ was recently found to have GTPase activity [51], however, it interacts with at least four proteins and is a component of the complex divisome [52]. From the point of view of gene product regulation, FtsZ concentrations dictate the initiation of cell division through interaction with FtsA and FtsQ [53]. In contrast, FabI and MurA interact with fewer proteins [5] possibly because they catalyze specific reactions. Also, FabI is the catalytic end point in fatty acid elongation and is subject to feedback inhibition by acyl-ACP in the fatty acid elongation cycle [54], and MurA is the catalytic starting point in peptidoglycan assembly [27] and may be regulated through feedback inhibition by UDP-N-acetylmuramic acid, a downstream product in the pathway [55]. Hence, it can be argued that changes in ACP or FtsZ levels are less tolerated than changes in FabI and MurA levels.

Specific control of single gene expression within a bacterial operon remains a challenge due to the polycistronic nature of transcripts. Indeed, antisense PNAs targeting genes of the *lac* operon have polar effects on transcript stability and translation [39]. To avoid this problem, we selected genes that are less likely to be affected: *fabI* and *murA* are predicted by RegulonDB (regulondb.ccg.unam.mx) and EcoCyc (http://ecocyc.org/) to be individually transcribed, *ftsZ*, which is at the 3′ end of an operon [24], and *acpP*, which is transcribed either alone or with *fabF* [25], a non-essential gene involved in thermal regulation of fatty acid biosynthesis [56]. We note that the potency of the *acpP*-specific PNA may be due to inhibition of *fabF* expression as well, although this seems unlikely, as it has been reported that tandem mutation in another gene (*fabB*) is needed for growth inhibition [56], and anti-*acpP* effects are rescued by plasmid *acpP* transcomplementation [37].

In summary, our results show essential genes are differentially required and their requirement level appears to reflect the extent of predicted protein-protein interactions. Both PNA and expressed antisense gene silencing methods gave similar results, despite having different inhibitory mechanisms. Therefore RNA silencing can be used to confirm and quantify essential gene requirement and serve as a molecular pre-screen, before investing in a search for specific inhibitors. It may also provide a useful tool for identifying reference genes for quantitative transcript profiling.

## Materials and Methods

### Bacteria and growth conditions for specificity testing of antisense agents

*E. coli* strain DH5α (Invitrogen) was transformed with recombinant plasmids pBAD-fabI, pBAD-acpP, pBAD-ftsZ and pBAD-murA (Table 2 and below) and clones were used for testing the specificity of gene silencing either by PNA treatment or antisense expression. Overnight *E. coli* cultures were standardized by OD_550_ readings to approximately 2×10^4^ cfu/ml. To test the specificity of antisense PNA, clones were grown in Mueller-Hinton broth (MHB; Oxoid) supplemented with ampicillin (100 µg/ml) and 4 µM of gene-specific antisense PNA in aqueous solution, either with or without 0.02–0.2% L-arabinose (Sigma) for induction of essential gene over-expression. Concentrations of L-arabinose were optimized for each clone.

To test expressed antisense specificity, clones containing both essential gene over-expressing plasmid and antisense-expressing plasmid (see below) were grown in MHB supplemented with ampicillin (100 µg/ml) plus chloramphenicol (30 µg/ml) and 0.1–75 mM IPTG (for induction of expressed antisense), either with or without 0.02–0.2% L-arabinose for induction of essential gene over-expression. Concentrations of IPTG and L-arabinose were optimized for each clone. Bacterial cultures were grown in a Bio-Tek PowerWave X340 spectrophotometer at 37°C with agitation every 5 min in 200 µl volumes in a 96-well plate. Growth was monitored by OD_550_ readings every 5 min. Each experiment was performed in triplicate.

### Plasmids and strains used for testing specificity of antisense agents

The ORFs of the four essential genes were cloned into the multiple cloning site (MCS) of pBAD/HisA vector (Invitrogen), allowing gene over-expression from the L-arabinose-inducible promoter. Sequences were amplified from K12 genomic DNA using primers acpP-OF/R, ftsZ-OF/R, murA-OF/R and fabI-OF/R, specific for *acpP*, *ftsZ*, *murA* and *fabI*, respectively (Table S1). Amplicons were digested with *NcoI* and *XhoI* (Fermentas) and cloned into the pBAD/HisA vector containing similarly digested ends. Recombinant plasmids were transformed into *E. coli* DH5α cells and selected on LB plates supplemented with ampicillin (100 µg/ml). Clones were then used for testing specificity of the PNA treatment. For testing specificity of the expressed antisense, clones containing either pBAD-fabI, pBAD-acpP, pBAD-ftsZ or pBAD-murA were further transformed with respective antisense-carrying plasmid, i.e. pHN682, pHNA, pHNZ or pHNM (Table 2 and below), and selected on LB plates supplemented with ampicillin (100 µg/ml) and chloramphenicol (30 µg/ml).

### Bacteria, growth conditions, PNA treatment and antisense expression for gene silencing

*E. coli* strain AS19 with a hyper-permeable phenotype was used in PNA treatments (total of 6 doses; Table 1) [40]. AS19 cultures in Mueller-Hinton broth (DIFCO) used for PNA (Panagene, Korea) treatments were prepared as described previously [57], and PNA concentrations (0–110 nM) were optimized for a titration in growth inhibition. TOP10 *E. coli* (Invitrogen) was used in transformation reactions of plasmids expressing antisense sequences (Table 2). Antisense expression was induced by various concentrations of IPTG (total of 6 doses) added to LB supplemented with chloramphenicol (30 µg/ml; Sigma). Overnight cultures of antisense-expressing clones were standardized by OD_550_ readings to approximately 2×10^5^ cfu/ml for IPTG induction. For antisense PNA treatment and IPTG induction, 20 µl of the appropriate PNA or IPTG concentration in aqueous solution was deposited into each well of a 96 well plate before the addition of *E. coli* in liquid culture to a final volume of 200 µl. Bacterial cultures were then grown in a VERSAmax spectrophotometer at 37°C with agitation for 5 s every 5 min. Growth was monitored by OD_550_ readings every 5 min and all cultures were harvested when the untreated control increased in turbidity of ∼0.1. Wells containing the same treatment were pooled at harvest and cells were pelleted for RNA extraction. Growth rate was calculated as ΔOD/Δt.

### Design of antisense PNA for gene silencing

Antisense PNA specific for *acpP* (Ec108), *fabI* (Ec107), *ftsZ* (Ec326) and *murA* (Ec330) were designed using optimal parameters for gene silencing [37], [38] (Table 1). Briefly, these parameters are: 9–12 mer PNA of an antisense sequence targeting the region of −10 to +10 around the start codon and conjugated to a cell wall-permeating peptide (KFF)_3_K. Candidate antisense sequences within the stated start codon region were checked for sequence uniqueness in similar regions using an *E. coli* database (http://genolist.pasteur.fr/Colibri/) before testing antisense inhibition of growth and gene expression, as described above and below.

### Plasmid expressing antisense for gene silencing

Antisense sequences were chosen based on length, location (120–160 nt around the start codon to include promoter and coding regions) [12] and for minimal secondary structures, which were predicted by RNAfold (http://www.tbi.univie.ac.at/RNA/RNAfold.html). Antisense sequences were cloned into the MCS of pHN678, which is flanked by a 38 bp paired-termini to stabilize antisense RNA inserts [12]. Antisense sequences of essential genes were amplified from K12 genomic DNA with primers acpP-XF1/R2, ftsZ-XF5/R5 and murA-XF/R specific for *acpP*, *ftsZ* and *murA*, respectively (Table S1). Amplicons were then digested with *Nco*I and *Xho*I and cloned into pHN678 with similarly digested ends. Recombinant plasmids were transformed in TOP10 cells and selected on LB supplemented with chloramphenicol (30 µg/ml). The *fabI* antisense expressing plasmid pHN682 has been described [12].

### Fluorescence microscopy

*E. coli* AS19 cultures were either left untreated or treated with sub-inhibitory (dose 3) and inhibitory (dose 6) concentrations of PNA and then viewed by fluorescence microscopy. Similarly, *E. coli* TOP10 cultures either left uninduced or induced with IPTG for moderate (dose 3) or high expression (dose 6) of antisense sequences were viewed microscopically. Cells were grown and harvested as described above, where 1 ml of cells were pooled and pelleted. Cells were washed once in an equal volume of 1×PBS, suspended in 100 µl of 1×PBS, and stained with DAPI (1 µM) for 5 min. Cells were viewed at 1000×magnification in a Leica DMRA2 microscope and images were captured and processed using Openlab software version 3.1.4.

### RNA extraction, reverse transcription and qPCR

Extraction of RNA from bacterial cells, followed by DNase I treatment were carried out using the RiboPure Bacteria Kit (Ambion) according to the manufacturer's protocol. RNA (200–500 ng) was converted to cDNA in a 25 µl reaction consisting of 1×RT reaction buffer, 5.5 mM MgCl_2_, 0.5 mM of each dNTP, 2.5 µM random hexamers, 0.4 U/µl RNase inhibitor, and 1.25 U/µl MultiScribe Reverse Transcriptase (Applied Biosystems). Each 25 µl of PCR reaction contained 12.5 µl of SYBR Green PCR buffer (Eurogentec), 100 nM of each primer (Biomers) and 5 µl of cDNA. Relative qPCR was carried out with primers acpP-F/R, fabI-F/R, ftsZ-F/R, murA-F/R specific for *acpP*, *fabI*, *ftsZ* and *murA*, respectively, as the target gene, and primers rpoA-F/R specific for *rpoA* as the reference gene (Table S1). Target and reference primer pairs were validated for amplification efficiencies for real time data analyses, either by the 2^-ΔΔCT^ method [58] for primers with similar efficiencies, or the 2^ΔCT (target)^/2^ΔCT (reference)^ method for primers with different efficiencies [59] for relative qPCR. Quantitation of *acpP*, *fabI*, *ftsZ* and *murA* mRNA was normalized against *rpoA* mRNA and calculated relative to the untreated sample. Mean values with error bars, representing standard deviation from three experimental replicates, were plotted against PNA or IPTG doses.

### MTL_50_ calculation

For each gene, data points from ΔOD/Δt per PNA or IPTG concentration (Figure 2) were plotted against relative mRNA per PNA or IPTG concentration (Figure 2) on a new graph. This results in a curve of relative ΔOD/Δt against relative mRNA for each gene, from which the MTL_50_ is estimated. The x-axis value where the curve intersects y = 0.5 (for 50% reduction in growth) gives the MTL_50_ value. As each experiment was carried out in triplicate, each gene had three curves from PNA treatment and three curves from expressed antisense experiments. For simplicity, only mean data resulting in an average curve for each gene is shown in Figure 3. Triplicate MTL_50_ values, estimated from triplicate curves, were used for statistical analysis in Figure 6.

### Statistical analysis

Statistical analyses of R^2^ for correlation, mean±SD and ANOVA were carried out using Excel. Tukey HSD Test for post-ANOVA comparisons was calculated at http://faculty.vassar.edu/lowry/hsd.html

## Supporting Information

Table S1 Primers used in this study.
(0.06 MB DOC)
