# Supplementary material for: Concurrent Growth Rate and Transcript Analyses Reveal Essential Gene Stringency in Escherichia coli
Source: PLoS One. 2009 Jun 26;4(6):e6061. doi: 10.1371/journal.pone.0006061 (PMC2698124; doi:10.1371/journal.pone.0006061)
Supplement: Table S1 — Primers used in this study. (0.06 MB DOC) [file pone.0006061.s001.doc]

Table S1. Primers used in this study

| Primer | Target | Primer sequence* (5’ -> 3’) | Size of amplicon (bp) | Purpose of amplicon |
| --- | --- | --- | --- | --- |
| acpP-OF | *acpP* | gaccatgggcactatcgaagaacgcgttaa | 323 | Cloning of *acpP* ORF into pBAD, resulting in pBAD-acpP |
| acpP-OR |  | gtcctcgagggagggaaaaaatg |  |  |
| ftsZ-OF | *ftsZ* | aaccatggttgaaccaatggaacttaccaat | 1171 | Cloning of *ftsZ* ORF into pBAD, resulting in pBAD-ftsZ |
| ftsZ-OR |  | aactcgagtcaattcttaatcagcttgcttac |  |  |
| murA-OF | *murA* | ctccatggataaatttcgtgttcaggggc | 1276 | Cloning of *murA* ORF into pBAD, resulting in pBAD-murA |
| murA-OR |  | gcctcgagacgattattcgcctttcacacg |  |  |
| fabI-OF | *fabI* | agccatgggttttctttccggtaagcgc | 810 | Cloning of *fabI* ORF into pBAD, resulting in pBAD-fabI |
| fabI-OR |  | acctcgagaacgattatttcagttcgagttc |  |  |
| acpP-XF1 | Antisense of *acpP* | cgccatggaagaagcattgttggtaacttc | 143 | Cloning of *acpP* antisense into pHN678, resulting in pHNA |
| acpP-XR2 |  | gcctcgaggaaaaccatcgcgaaag |  |  |
| ftsZ-XF5 | Antisense of *ftsZ* | cgtccatggcagcattaccgccgccgc | 147 | Cloning of *ftsZ* antisense into pHN678, resulting in pHNZ |
| ftsZ-XR5 |  | tgtctcgaggaggccgacgatgattacggc |  |  |
| murA-XF | Antisense of *murA* | cgtccatgggagcagcatttttagcgc | 148 | Cloning of *murA* antisense into pHN678, resulting in pHNM |
| murA-XR |  | tgtctcgaggctatgggcgattcgc |  |  |
| acpP-F | *acpP* | caggaagaagttaccaacaatgctt | 72 | qPCR of *acpP* |
| acpP-R |  | ccagctcaacggtgtcaaga |  |  |
| fabI-F | *fabI* | tgccgctccatgctgaa | 59 | qPCR of *fabI* |
| fabI-R |  | tcagcgccaaggtaggaaa |  |  |
| ftsZ-F | *ftsZ* | agcagaagccggttgctaaa | 61 | qPCR of *ftsZ* |
| ftsZ-R |  | tccggctctttcgcagtt |  |  |
| murA-F | *murA* | cactacctgcgcgttgtacga | 64 | qPCR of *murA* |
| murA-R |  | tgttcgaggccagaaatgtg |  |  |
| rpoA-F | *rpoA* | aagctggtcatcgaaatggaa | 65 | qPCR of reference gene |
| ropA-R |  | gccgcacgacgaatcg |  |  |

*Restriction sites are underlined.
